# Balkan Medical Journal and Legal Regulation

**DOI:** 10.4274/balkanmedj.galenos.2019.2019.2.002

**Published:** 2019-02-28

**Authors:** Mustafa İnan, Hakan Erbaş, Zafer Koçak, Cem Uzun

**Affiliations:** 1 Department of Pediatric Surgery, Trakya University School of Medicine, Edirne, Turkey; 2 Department of Biochemistry, Trakya University School of Medicine, Edirne, Turkey; 3 Department of Radiation Oncology, Trakya University School of Medicine, Edirne, Turkey; 4 Department of Otolaryngology, Trakya University Faculty of Medicine, Edirne, Turkey

Balkan Medical Journal, the official university/academic journal of Trakya University Faculty of Medicine, is under the ownership of the deanery of the faculty and aims to propagate the current medical scientific knowledge in the Balkan region. The rectorate of Trakya University logistically supports the journal. On the other hand, the editor-in-chief is scientifically independent and cannot be pressurized or manipulated in any way regarding the scientific contents and operation of the journal. Nonetheless, the editor-in-chief has obligations toward the journal owner and editorial board. His/her editorial independence, rights, and responsibilities are aptly described and documented by the international scientific publishing communities. These include the editor’s responsibilities, autonomy, and accountability of the journal’s scientific content (1,2,3,4,5). Besides the editor-in-chief, the other editorial team members (managing, deputy, or assistant editors or any other editorial board member), the publisher, and the owner of a journal also have responsibilities, rights, and duties, which should all be documented in the journal legal regulation. Implementation of journal legal regulation will guarantee independent and responsible management of the journal, protect the editorial freedom, and ensure the team’s transparency at work.

Since its establishment, the Balkan Medical Journal has aimed to select and publish distinctive, original articles in general medicine to contribute to universal scientific knowledge and to share its experience through educational activities for authors, reviewers, and editors (6). Despite the editor-in-chief and members of the editorial board getting replaced after their tenures, the Balkan Medical Journal’s management has always believed that the fulfillment of its mission depends on the fundamental conditions of ethical values, respect, institutional attitude, and teamwork (7). The effort to maintain the editorial freedom has posed distinct challenges arising from regional dynamics in the Balkan region, as in any part of the world (7,8,9,10). Becoming an official member of important international publishing organizations, such as International Council of Medical Journal Editors (ICMJE), the World Association of Medical Editors (WAME), the Council of Science Editors (CSE), and the European Association of Science Editors (EASE), in addition to being formally recognized by the Committee on Publication Ethics (COPE), were all important milestones that have enabled Balkan Medical Journal to develop, maintain, and guarantee its editorial freedom (10).

A careful review of the literature reveals that the Balkan region has taken permanent steps towards institutionalization with the successful publication practices of journals. Croatian Medical Journal stands out as the most significant journal in this regard. A paper by Marusic et al. (11) in 2005 demonstrates that the Croatians have combined the dynamics of four universities in their country to create a journal of national value and have shared their developmental experiences. Undoubtedly, the significant cornerstone in the success attained by the Croatians is the journal’s legal regulation, which documents consensus.

For a journal to be successful in the international arena, reasonably organized managerial processes are required. Clear definitions of work packages, workflows, duties and responsibilities, and assignment and dismissal criteria are essential for the transparency of managerial processes. The journal’s owner, editor-in-chief, editorial board, publisher, and authors effectively communicating with each other within the framework of rules will prevent chaotic and destructive conflicts that may harm the reputation and its mission. Furthermore, the inscription of such rules promotes scientific autonomy of the editor and prevents misuse of editorial freedom thereby resulting in the continuous success of a journal.

The efforts to establish a legal regulation, an agreement between the owner and the editor, and an ombudsman began approximately five years ago for Balkan Medical Journal. A draft text of legal regulation was submitted for discussion to the editorial board at that time. Intensive efforts have been carried out to achieve a certain degree of maturity. Since no other university/academic general medicine journal in Turkey is also indexed in internationally published SCI-E and Medline, it took time for all stakeholders to draft a consensus document. Thanks to the previous editor-in-chief of Balkan Medical Journal and the current vice-rector of Trakya University responsible for scientific journals (Cem Uzun), who has encouraged all editors of Trakya University journal to establish legal regulations, we have a transparent scientific publishing atmosphere to discuss, write, and own individual legal regulation for each journal. Throughout this process, the journal’s management has endeavored to consult the opinions of the journal’s owner and the university legal regulation committee to establish a legal regulation for the journal that is ethical, practical, sustainable, and conforms to the national laws and international scientific values. We believe the journal legal regulation, finalized in January 2019 and presented below, serves as a role model for all scientific circles and the Balkans and will contribute to the advancement of institutional scientific publications of medicine in the region. Nonetheless, this legal regulation is open to being updated and improved whenever needed, as our other policies. The journal’s editorial board extends its gratitude to all current and past employees, the Dean’s Office of the Faculty of Medicine of Trakya University, and to the current rectorate of Trakya University for their valuable contributions and encouragement to building the development of the journal legal regulation to protect editorial freedom and provide successful journal management of all Trakya University scientific journals.
